# Frustration Dynamics and Electron-Transfer Reorganization Energies in Wild-Type and Mutant Azurins

**DOI:** 10.1021/jacs.1c13454

**Published:** 2022-02-16

**Authors:** Xun Chen, Mingchen Chen, Peter G. Wolynes, Pernilla Wittung-Stafshede, Harry B. Gray

**Affiliations:** † Center for Theoretical Biological Physics, Houston, Texas 77005, United States; ‡ Department of Chemistry, Rice University, Houston, Texas 77005, United States; § Department of Biosciences, Rice University, Houston, Texas 77005, United States; ∥ Department of Biology and Biological Engineering, Chalmers University of Technology, 412 96 Gothenburg, Sweden; ⊥ Beckman Institute and Division of Chemistry and Chemical Engineering, California Institute of Technology, Pasadena, California 91125, United States

## Abstract

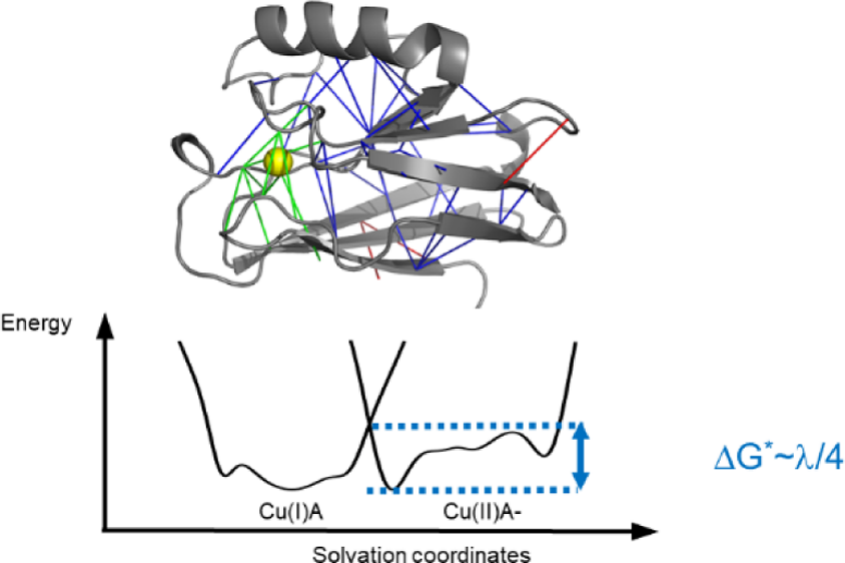

Long-range electron tunneling through metalloproteins is facilitated by evolutionary tuning of donor–acceptor electronic couplings, formal electrochemical potentials, and active-site reorganization energies. Although the minimal frustration of the folding landscape enables this tuning, residual frustration in the vicinity of the metallocofactor can allow conformational fluctuations required for protein function. We show here that the constrained copper site in wild-type azurin is governed by an intricate pattern of minimally frustrated local and distant interactions that together enable rapid electron flow to and from the protein. In contrast, sluggish electron transfer reactions (unfavorable reorganization energies) of active-site azurin variants are attributable to increased frustration near to as well as distant from the copper site, along with an exaggerated oxidation-state dependence of both minimally and highly frustrated interaction patterns.

## 1 Introduction

**Figure 1:**
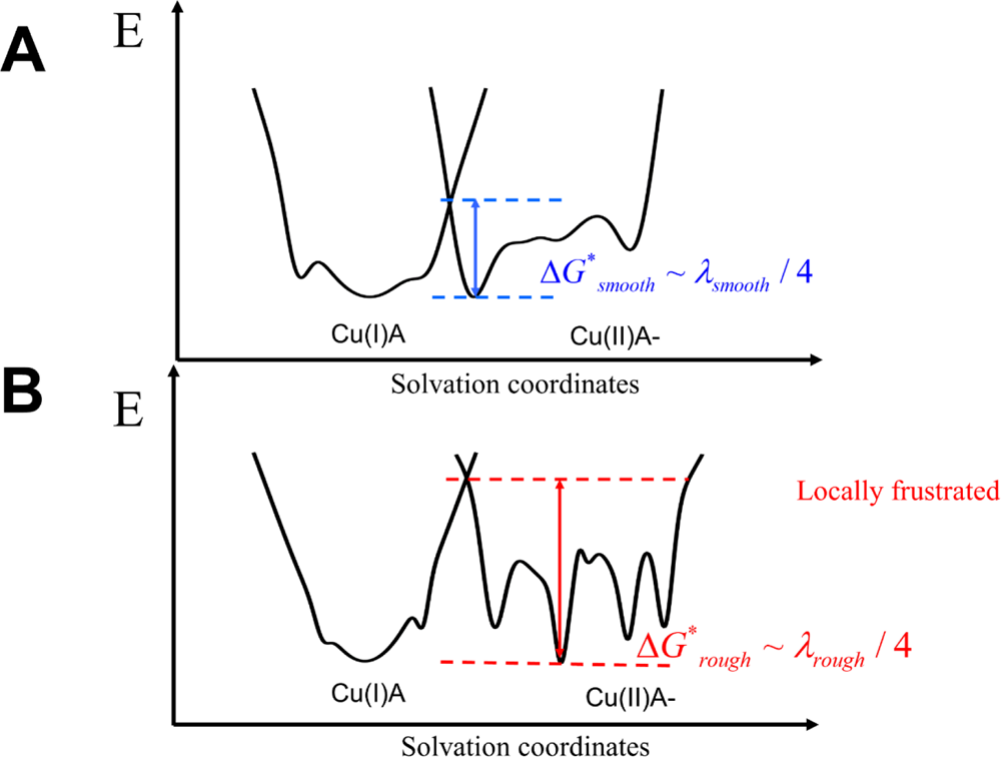
Electron transfer in proteins relies on the nonadiabatic crossing of two potential landscapes: one for the reactant and the other for the product. We show 1D schematics of the two energy landscapes for charge reorganization in azurin when coupled to the electron acceptor A, which is tuned to make the reaction energetically neutral. Panel (A) shows the plot for reorganization energy on the relatively smooth, minimally frustrated landscape of wild-type (WT) azurin, while (B) refers to the reorganization on the more flexible, locally rugged landscape with more frustrated interactions of mutated azurins. The barrier for the electron transfer reaction is roughly one-fourth of the reorganization energy on an equivalent smooth harmonic surface. In all the azurins studied, the Cu(I) surface shows somewhat less frustration than Cu(II).

Biological electron transfer reactions, the core of the cell’s energy economy, generally require the reorganization of the protein environment surrounding a metal ion or a co-factor.^1−4^ The specificity of the structural fold enables the evolutionary tuning of thermodynamic reduction potentials, both directly, through constraining the local coordination geometry of the metal with specific amino acid ligands and through electrostatic interactions with more distant residues. The rate of electron transfer depends on the barrier for reorganizing the protein environment, which involves changing from one protein conformation, solvating the initial charge state of the ion, to the one solvating the final state. This reorganization energy is not determined by the single structure of the protein but by the energy landscape of available protein conformations solvating the co-factor. A very rigid protein environment could hold the active site near the transition state for electron transfer (a minimal reorganization barrier, Figure 1A). On the other hand, a floppy protein environment, like one in a polar liquid solvent, will allow access to many configurational states and could make the environmental reorganization more costly, disfavoring electron transfer (Figure 1B).^5^ Tuning the local energy landscape to control rates for conversion between protein configurational states provides a further possibility of evolutionary refinement beyond the regulation of thermodynamic stabilization of folded protein structures. The folding of a floppy polypeptide to a specific global structure requires the harmonious cooperation of many interactions within the protein. Minimizing the conflicts between different interactions, called frustration,^6^ allows the protein to globally fold efficiently on a funneled energy landscape.^7^ Achieving foldability in evolution, however, does not require the complete elimination of frustration. Some locally frustrated interactions can be tolerated by evolution but at the cost of proliferating thermally accessible substrates on the energy landscape of the functional protein. Such local frustration substantially increases active-site redox reorganization energies, turning off the distant electron tunneling reactions required for function.

In this paper, we explore how tuning the frustration of the energy landscape links to the electron flow in azurin, a well-studied blue copper protein.^8−21^ To quantify and locate sites of conflicting interactions in azurin, we use atomistic frustration analysis, based on algorithms inspired by energy landscape theory.^6,7^ This algorithm compares the energies of individual interactions in a specific protein with the statistics of the energies of virtual mutants in which, computationally, alternate amino acids are locally introduced and/or local structural changes are imposed on the structure. Using this algorithm, interactions in the original protein that are considerably more stable than those found in these variants are classified as “minimally frustrated”—they provide the dominant driving force for folding to the specific native structure by building the funneled landscape. Many interactions in the original protein turn out to be neutral according to this algorithm, but often a conflicting evolutionary constraint is revealed, such as the need for formation of a binding site or a flexible site for allostery, which forces a part of the protein to be frustrated. We here explore the influence of frustration on the electron transfer function of wild-type WT azurin and three active-site mutants, C112D, C112D/M121E (pH 7.0 and 9.0), and C112D/M121L azurin variants.^4,11,15,18,19^

Of relevance is that Zaballa et al. have shown by solution NMR that residues in the surroundings of the copper-binding pocket of apo azurin are not as rigidly fixed as in the holo protein.^22^ In accordance with that finding, our frustration analysis of the apo protein (using crystal structure data) shows that the flexibility detected in NMR correlates with more highly frustrated interactions and fewer minimally frustrated interactions for those residues (Figure S1). Upon comparison, the frustration analysis reveals fewer minimally frustrated interactions in total for apo azurin as compared to holo forms with Cu(I) or Cu(II) in the copper site (Figure S2). Moreover, neither the apo nor the holo wild-type (WT) protein has any highly frustrated interactions in the copper-binding site (Figure S3). Importantly, it has been established that the binding site “rack effect,” here called “constrained coordination,”^6^ tunes the Cu(II)/Cu(I) formal potential and lowers the reorganization energy of the WT protein to provide optimal function.^1−4,12,13,17^ The reorganization energy of C112D is well above that [0.7(1) eV] of WT.^15^ Here, we report detailed frustration analyses that shed new light on residue–residue interactions that tune the redox properties of WT and mutant azurins.

## 2 Results and Discussion

**Figure 2:**
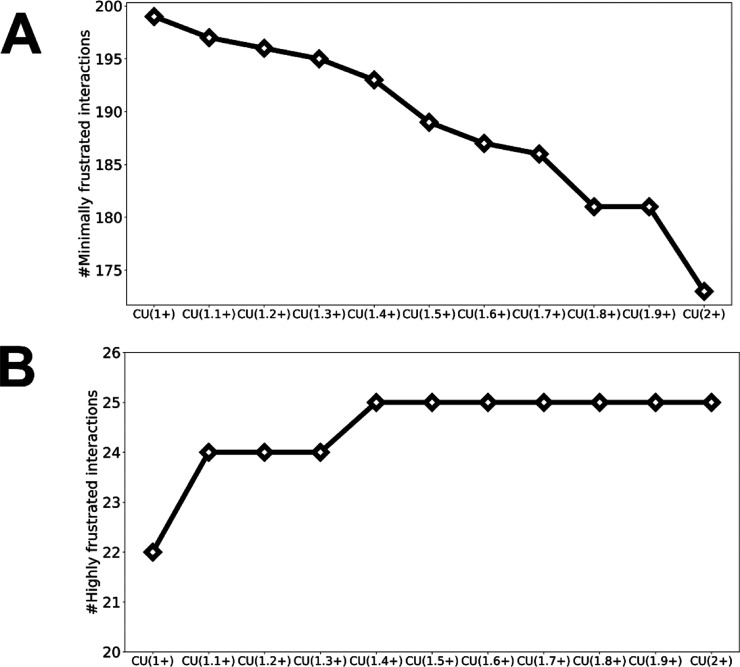
Number of frustrated interactions in WT azurin as the charge is interpolated on a model tetrahedral copper center. This partial charging strategy reflects the Marcus construction of a polarizable environment. (A) Number of minimally frustrated interactions. (B) Number of highly frustrated interactions.

Years of theoretical and experimental work have established that a relatively high reduction potential and low electron-transfer reorganization energy of azurin are attributable to a constrained inner-sphere copper geometry.^8,21,23,24^ Copper coordination in azurin can be described as distorted trigonal pyramidal (Figure S4), which is a compromise between the preferred coordination geometries for Cu(I) and Cu(II), but it is believed to favor Cu(I).^4^ In accordance with this long-held view, the number of minimally frustrated interactions throughout the protein decreases as the charge on the central metal increases, since the trigonal pyramidal coordination geometry (tetrahedral in our model) favors Cu(I). As would be expected, the number of highly frustrated interactions throughout the protein is greater when the copper is oxidized than when it is reduced. Results of the calculations of both minimally and highly frustrated interactions (i.e., the total number of such interactions in the whole protein) as a function of charge (going from +1 to +2 in increments of 0.1) on a tetrahedrally coordinated copper in WT azurin are shown in Figure 2.

**Figure 3:**
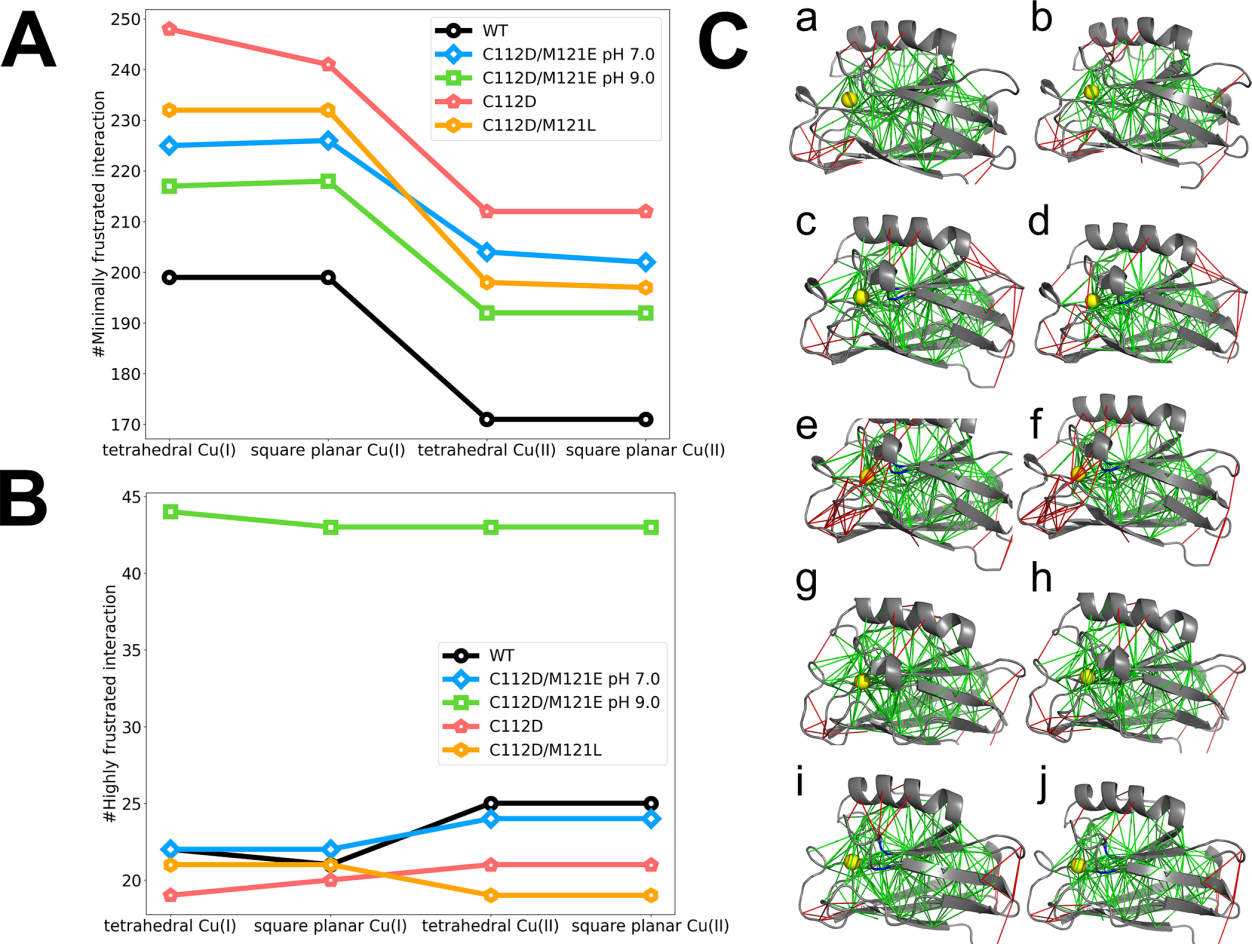
Number of frustrated interactions in WT and mutant azurins on model tetrahedral and square planar copper centers. (A) Number of minimally frustrated interactions. (B) Number of highly frustrated interactions. Colors: WT, black; C112D/M121E at pH 7.0, blue; C112D/M121E at pH 9.0, green; C112D, red; and C112D/M121L, orange. (C) Atomistic frustration patterns in WT and mutant azurins. (a) Tetrahedral Cu(I) WT azurin. (b) Square planar Cu(II) WT azurin. (c) Tetrahedral Cu(I) C112D/M121E at pH 7.0. (d) Square planar Cu(II) C112D/M121E at pH 7.0. (e) Tetrahedral Cu(I) C112D/M121E at pH 9.0. (f) Square planar Cu(II) C112D/M121E at pH 9.0. (g) Tetrahedral Cu(I) C112D. (h) Square planar Cu(II) C112D. (i) Tetrahedral Cu(I) C112D/M121L. (j) Square planar Cu(II) C112D/M121L. Colors: protein, gray; Cu, yellow sphere; minimally frustrated interactions are shown as green lines; and highly frustrated interactions are shown as red lines.

Comparisons of frustration patterns for WT and active-site mutant azurins are instructive. In our analysis of the frustration patterns throughout the proteins, we modeled tetrahedral and square planar geometries for both Cu(I) and Cu(II) redox states (Figure 3). Note that the starting points for the Cu(II) forms of proteins were taken from crystal structures. Interestingly, the copper oxidation state is the main factor affecting the frustration landscape, whereas the inner copper coordination structure does not have much influence on the interaction dynamics.

The pattern of minimally frustrated interactions throughout the three active-site mutant proteins (C112D, C112D/M121E, and C112D/M121L azurins) is the same as in WT, albeit with more such interactions in the mutants in both redox states (Figure 3A,B). The large decrease in minimally frustrated interactions accompanying hole transfer to Cu(I) azurins is apparent in all variants. The number of highly frustrated interactions is lower in mutants C112D and C112D/M121L than in WT azurin, consistent with a less constrained overall fold. Of interest is the increase in the number of highly frustrated interactions in C112D/M121E (pH 9.0), whose crystal structure reveals tortured outer-sphere copper coordination^19^ but an overall similar fold. In contrast, this variant at pH 7.0 shows a WT pattern of highly frustrated interactions throughout the folded protein.

Structural portraits showing both minimally frustrated (green lines) and highly frustrated (red lines) interactions in Cu(I) and Cu(II) forms of WT and mutant azurins are displayed in Figure 3C. Minimally frustrated interactions occur throughout the proteins in both active-site coordination geometries, and many of the highly frustrated interactions occur in distant (from the Cu) regions of the protein. Notably, there are variations in the positions of both highly and minimally frustrated interactions distant from the copper site when comparing WT with the mutants. This finding indicates that specific networks of long-range couplings involving highly as well as minimally frustrated interactions distant from the WT active site stabilize the constrained copper coordination. Perturbing constrained copper coordination by nearby mutations affects the pattern of interactions both near to and distant from the copper in the protein fold. Notably, many more highly frustrated interactions near the active site of the C112D/M121E (pH 9.0) mutant are clearly visible in Figure 3C(e,f).

Taken together, the mutants have more minimally frustrated interactions than WT in total, but WT has more (or similar numbers of) highly frustrated interactions than the mutants (except for C112D/M121E at pH 9.0). This likely reflects the evolutionary cost of creating a protein with constrained metal coordination. In other words, to evolve locally constrained coordination around copper, as needed for function, it appears that some frustrated interactions within the fold have to be accepted. Interestingly, this finding indicates that the mutants would exhibit less rugged folding landscapes than WT.

**Figure 4:**
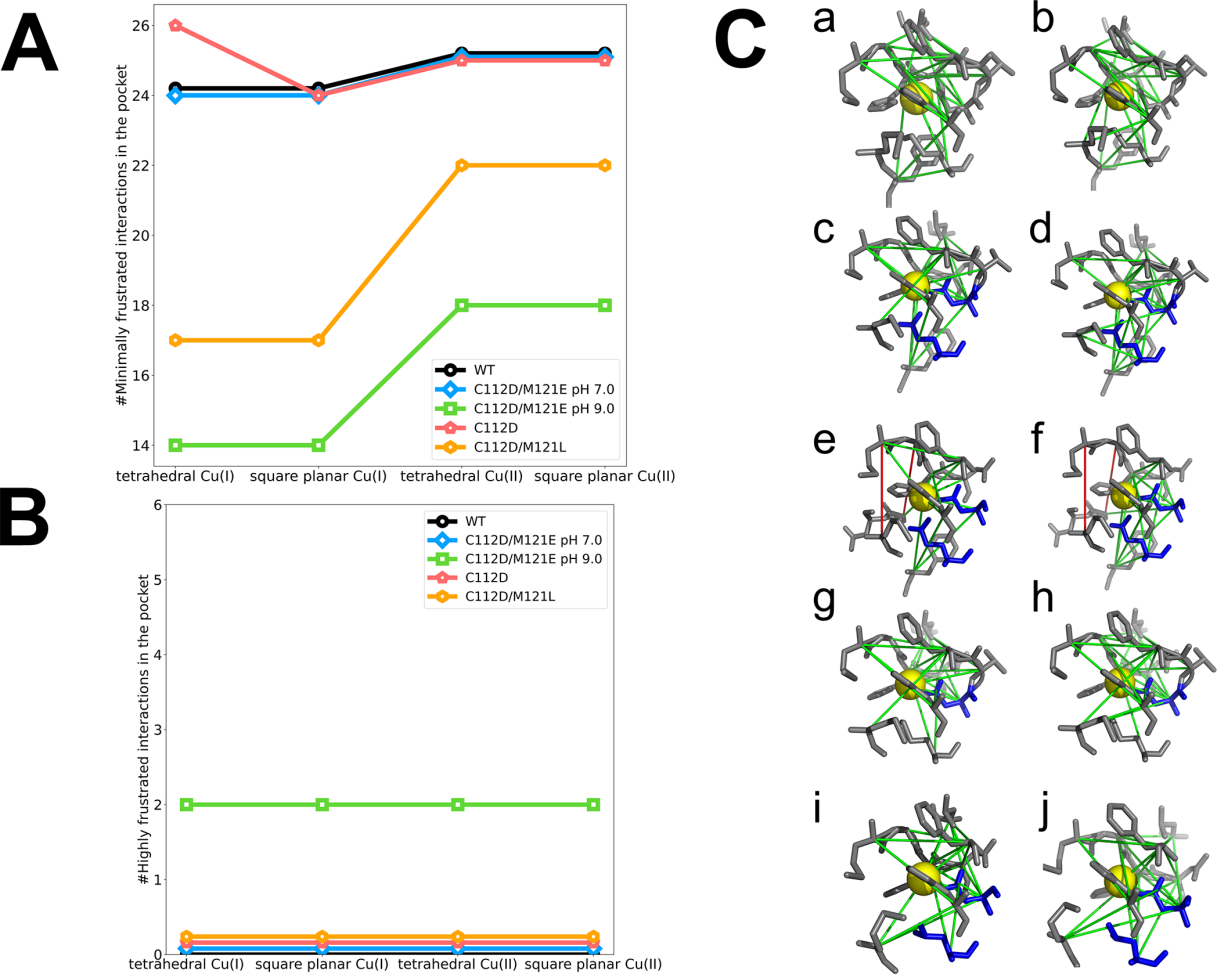
Number of frustrated interactions within 6 Å of copper for tetrahedral Cu(I) and square planar Cu(II) WT and mutant azurins. (A) Change in the number of minimally frustrated interactions in the binding pocket (the black WT line overlaps that of C121D/M121E at pH 7.0). (B) Change in the number of highly frustrated interactions in the binding pocket. Colors: WT, black; C112D/M121E at pH 7.0, blue; C112D/M121E at pH 9.0, green; C112D, red; and C112D/M121L, orange; (note that the black (WT), blue (C112D/M121E at pH 7.0), and red (C112D) lines overlap those of C112D/M121). (C) Binding pocket frustration patterns for WT and mutant azurins. (a) Tetrahedral Cu(I) WT. (b) Square planar Cu(II) WT. (c) Tetrahedral Cu(I) C112D/M121E at pH 7.0. (d) Square planar Cu(II) C112D/M121E at pH 7.0. (e) Tetrahedral Cu(I) C112D/M121E at pH 9.0. (f) Square planar Cu(II) C112D/M121E at pH 9.0. (g) Tetrahedral Cu(I) C112D. (h) Square planar Cu(II) C112D. (i) Tetrahedral Cu(I) C112D/M121L. (j) Square planar Cu(II) C112D. Colors: binding pocket, gray; Cu, yellow sphere; mutated residues, blue. Minimally frustrated interactions are shown as green lines and highly frustrated interactions as red lines.

We next focused on the interactions within 6 Å of the Cu-binding pocket (Figure 4A,B), allowing comparison of local frustration interactions with those distant from copper among the azurin variants. Residues in this region having donor atoms making direct bonds to copper are in inner-sphere coordination, whereas those in van der Waals and hydrogen bonding contact are in outer-sphere coordination. Of interest, the number of minimally frustrated interactions within 6 Å is constant in going from Cu(I) to Cu(II) in WT azurin (Figure 4A), indicating that the changes seen in those interactions for the whole protein (Figure 3A) occur outside the binding pocket. Also, both ligated Cu(I) and Cu(II) in C112D and the C112D/M121E (pH 7.0) variants, respectively, display similar levels of minimally frustrated interactions within 6 Å of the active site as found in the WT protein. The situation is different in the C112D/M121L and C112D/M121E (pH 9.0) variants, where the number of minimally frustrated interactions near the active site is lower in the Cu(I) than in the Cu(II) protein and lower than those seen in WT and the other variants in both Cu(I) and Cu(II) oxidation states. This observation supports the view that the mutations introduced in these two variants create a binding site that favors Cu(II) over Cu(I).^9,18,19^ As expected for the tortured outer-sphere coordination in C112D/M121E (pH 9.0) azurin, there are two highly frustrated interactions within 6 Å (Figure 4B), but there are no such interactions in WT and other variants.

Minimally frustrated interactions within 6 Å of the Cu of WT and mutant azurins are marked in Figure 4C, as are the highly frustrated interactions in that region of C112D/M121E (pH 9.0). Interestingly, although WT as well as the C112D and C112D/M121E (pH 7.0) variants have similarly high numbers of minimally frustrated interactions within 6 Å, those interactions are not positioned at the same places when comparing the two copper oxidation states for each variant or when comparing the variants to each other. In striking contrast, the highly frustrated interactions in Cu(I) and Cu(II) C112D/M121E (pH 9.0) proteins are virtually identical, once again indicating that the Cu-binding pocket of this mutant is in a misfolded region of the WT energy landscape. We may conclude that to create a redox-active metalloprotein with low electron-transfer reorganization energy, there should be very few frustrated interactions within 6 Å of the metal-binding pocket along with very small changes in minimally frustrated interactions upon changes in the oxidation state.

**Figure 5:**
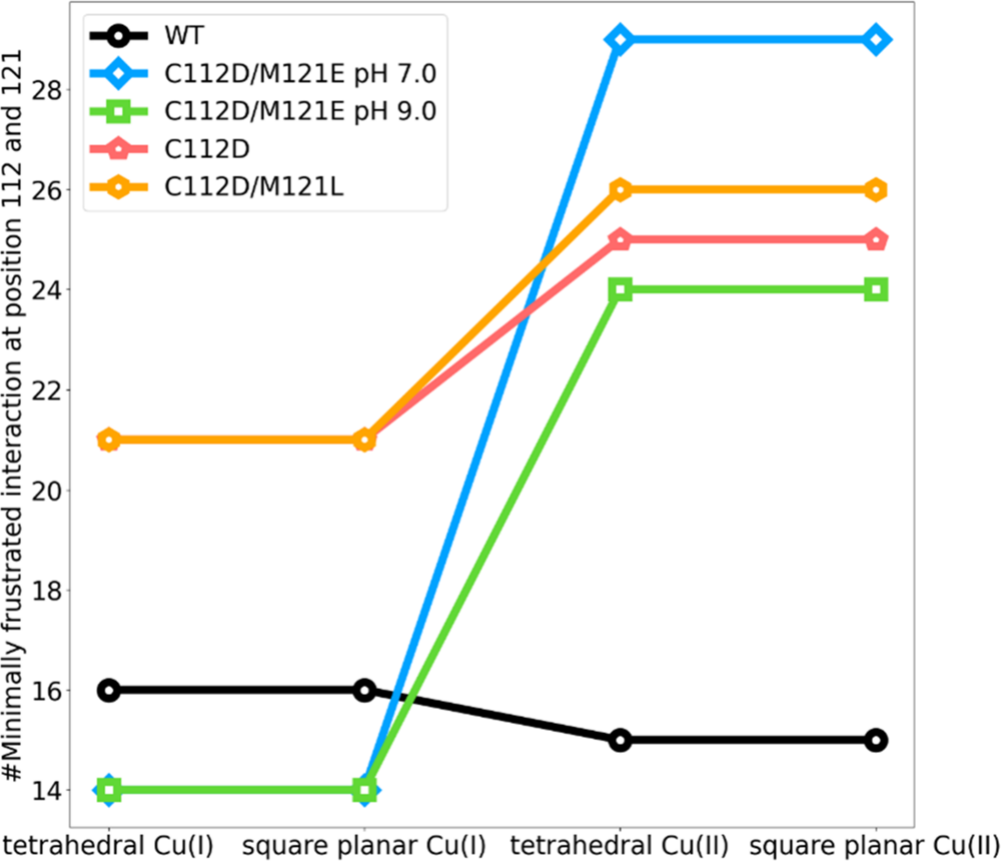
Number of minimally frustrated interactions at positions 112 and 121 in tetrahedral Cu(I) and square planar Cu(II) WT and mutant azurins, respectively. Colors: WT, black; C112D/M121E at pH 7.0, blue; C112D/M121E at pH 9.0, green; C112D, red; and C112D/M121L, orange.

In addition, we analyzed all short-range interactions with mutated residues D112 (for C112) and E121 or L121 (for M121). Minimally frustrated interactions with residues 112 and 121 are shown in Figure 5. In contrast to WT, where minimally frustrated interactions with these two residues do not depend on the copper oxidation state, all variants display more minimally frustrated interactions with the two mutated residues in Cu(II) than in Cu(I) proteins. Since the introduced residues favor interactions with Cu(II), our results support the view that ligation by native residues C112 and M121 is essential for functional Cu(II/I) electron flow.^8^

**Figure 6:**
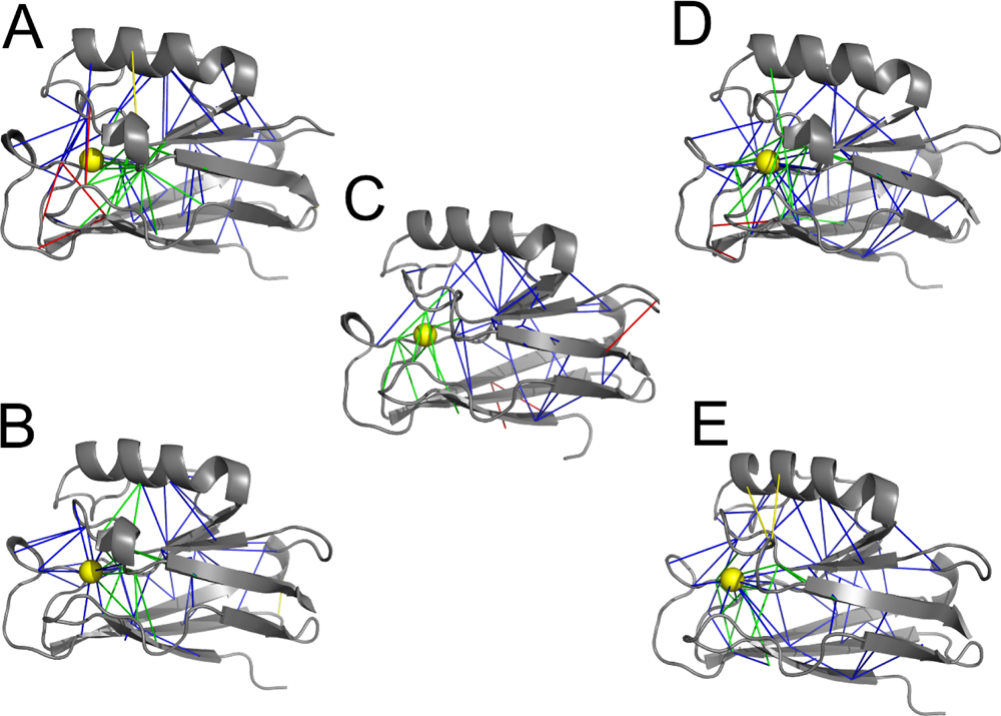
Changes in frustration patterns in going from tetrahedral Cu(I) to square planar Cu(II) coordination in mutant and WT azurins. (A) C112D/M121E at pH 7.0; (B) C112D/M121E at pH 9.0; (C) WT; (D) C112D; and (E) C112D/M121L. Protein, gray; Cu, yellow sphere; added minimally frustrated interactions from tetrahedral Cu(I) to square planar Cu(II) coordination, green lines; loss of minimally frustrated interactions, blue lines; added highly frustrated interactions, yellow lines; and loss of highly frustrated interactions, red lines.

As the key determinant for the low electron-transfer reorganization energy is constrained copper coordination, we directly compared changes in frustration patterns throughout the WT and mutant proteins upon copper redox change. The positions of changes in frustrated interactions when going from tetrahedral Cu(I) to square planar Cu(II) in these azurins are shown in Figure 6 (in the legend, we define four types of changes in frustration interactions, counted in Figure S5). Striking but perhaps not surprising is the appearance of highly frustrated interactions within 6 Å of copper upon electron removal in C112D/E121 (pH 7.0) azurin. The crystal structure of this mutant shows nonoptimal constrained copper coordination, as the potentially ligating E121 carboxylate oxygen can only make a very weak Cu···O(E121) bond in the Cu(II) state.^19^ Also interesting is the observation that there are many minimally frustrated interactions throughout all five proteins that become neutral (blue lines, Figure 6) upon Cu(I) oxidation. This finding supports the view that the constrained copper coordination in blue copper proteins favors Cu(I) over Cu(II).^4^ Inspection of the region around the copper site (Figure 6) shows WT to have more green lines (neutral interactions becoming minimally frustrated upon oxidation) and fewer blue lines (minimally frustrated interactions that become neutral upon oxidation) than in the mutants. Of interest are three highly frustrated interactions that appear upon copper oxidation in WT azurin. These interactions, which are placed distant from the copper site in the fold, are not observed upon copper oxidation in mutant proteins.

## 3 Concluding Remarks

For biology to exploit electron transfer processes, the evolutionary development of metalloproteins has been forced to acknowledge quantum mechanics as a selection constraint. The nonadiabatic nature of distant charge transfer^1−3^ requires the simultaneous sculpting of two protein energy landscapes: one for the protein with the reduced metal ion and another for the oxidized form. This sculpting involves the interplay of frustration in each of these landscapes. Evolving binding sites that are locally rigid entails further reducing protein frustration in the second shell around the metal ions beyond the level needed simply to fold. At the same time, some frustration in the primary coordination sphere of one of the charge states is typically needed to tune formal potentials. The frustration analysis of residue–residue interactions in azurin undertaken in this paper shows these quantum mechanical constraints on the landscapes at work.

We have shown that constrained trigonal pyramidal coordination, which lowers the Cu(II/I) reorganization energy of WT azurin, comes at the expense of frustrated interactions distant from the active site. Constrained inner-sphere coordination, which favors Cu(I), is relaxed in C112D and related mutants, where Cu(II) is not wedged into an energetically disfavored binding site.^11,15^ Unlike other C112D mutants, constrained coordination in C112D/M121E (pH 9.0) azurin is highly frustrated in both Cu(I) and Cu(II) redox states in accordance with crystal structure analysis.^19^

In the naturally evolved protein, the network of interactions that support copper inner-sphere coordination is tuned to give copper site rigidity and low electron-transfer reorganization energy. In the mutants, in contrast to WT, we found a few frustrated interactions in the copper-binding site that differ between the Cu(I) and Cu(II) states. It will be interesting to explore how general these observations are for other systems. Likewise, co-evolutionary analysis of families of electron transfer proteins may give us a path to quantify the strength of evolutionary constraints made necessary to facilitate biological electron transfer.

## 4 Methods

### 4.1 Energy Function Using the Atomistic Model Rosetta

To evaluate the energy of the native structure and its decoy structures, we use an atomistic forcefield, Rosetta.^25^ The Rosetta energy function has succeeded in protein structure prediction,^26^ protein structure refinement,^27^ and protein design.^28^ While coarse-grained force fields based on machine learning, such as AWSEM,^29^ are sufficient for understanding frustration in folding and protein–protein binding,^6,7,30−32^ the study of the role of ligands requires a fully atomistic description like Rosetta. Chen et al. have already used this force field to survey frustration in allosteric proteins and dimers as well as predict drug specificity.^33^ To generate the decoys of native structures, both the residue identities and locations in contacts were randomized, as was done previously using the AWSEM coarse-grained energy function. Then, after the protein sequence is randomly shuffled, the shuffled sequence is repacked onto the backbone, which remains unperturbed so as to allow only the sidechains to repack. To eliminate potential sidechain clashes, a short Monte Carlo relaxation is then performed. Following these steps, we obtain all the contact energies in the decoy ensemble *E*_ij_^U^ as well as the contact energies of the native sequence *E*_*ij*_^0^. Protein contacts are defined as having distances between C-α atoms of residues within a cutoff of 10 Å. Since in the paper we focus on changes of frustration as the charge is varied, we ensure that the same set of decoys is used for each charge state to avoid statistical fluctuation.

Because of the many-body construction of the Rosetta all-atom force field,^25^ the pairwise energy changes for interactions between residue *i* and residue *j*. *E*_*ij*_ are defined through total interaction energies and their changes when virtually mutating any of the two residues in contact.
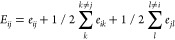
*e*_*ij*_ is the direct interaction between residue *i* and residue *j*. The terms 1/2∑_*k*_^*k*≠*j*^*e*_*ik*_ and 1/2∑_*l*_^*l*≠*i*^*e*_*jl*_ account for the auxiliary background interaction based on many-body effects. Here, we used the RET2015 version of the Rosetta energy function to compute the interaction energies.^25^

### 4.2 Definition of Local Frustration and Frustration Difference

The frustration index quantifies local frustration. The frustration indices are based on energy differences between the native conformation and its decoys. We gather the statistics of the local energy contributions by perturbing both the sequence and the local structure of the protein. The sequence space is randomly sampled according to the native amino acid frequency distribution as described above.^34^ Based on the recomputed energy of 300 appropriately distributed decoys for each contact, a histogram of the energy of these decoys can be constructed to compare with the native energy E_0_. The frustration index for the contact between residue *i* and residue *j* is defined as the *Z* score of energy of the native pair compared with the values for the corresponding decoys.
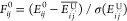


The pairwise interaction energy of the native conformation is defined as *E*_*ij*_^0^, and the pairwise interaction energy of the corresponding decoys is defined as *E*_*ij*_^U^. The frustration index is a site-specific measure of the energy fitness for folding the native contact compared to its alternatives and reflects the local energy difference between the native conformation and the average of its decoys normalized by the standard deviation σ(*E_i'j'_^U^*) of the energy distribution of decoys. A pair of contacts contributes to the folding funnel when its frustration index is sufficiently negative compared to other alternatives, while the other alternative structures are energetically accessible when the frustration index is positive. As discussed in Ferreiro et al.,^35^ the contacts are classified as being minimally frustrated, highly frustrated, or neutrally frustrated depending on their energetic *Z*-score value. In this work, the active-site copper is treated as a special residue. Contacts whose frustration index is more positive than 0.5 are identified as highly frustrated interactions, and contacts whose frustration index is more negative than −2.5 are identified as minimally frustrated interactions.

In this work, we explored the frustration differences between interactions in WT azurin and its mutants. When the frustration index of a pair in the WT protein is more positive than 0.5 but the mutant is below 0.5, we identify this contact as having a decreased level of highly frustrated interactions.

### 4.3 Copper Force Fields

Cu(I) prefers tetrahedral coordination, while four-coordinate Cu(II) is square planar.^25^ In standard Rosetta, the parameters of metal ions only acknowledge tetrahedral coordination. Therefore, we have introduced a distinct potential favoring square planar geometry, whose parameters are based on the structure of a salt of bis(1,10-phenanthroline)-Cu(II), CID 4375892.^36^ The radii of the two different copper oxidation states are based on averages found in crystal structures of azurin and its mutants. The parameters for square planar Cu(II) and tetrahedral Cu(I) potentials are in the Supporting Information.

### 4.4 Details of Frustration Analyses

In the frustration analyses, 300 shuffled sequences were used. In this work, the differences between different copper oxidation states in WT azurin and the differences between WT and mutants are small. To avoid fluctuations due to the use of different decoy sets of limited size, we used the same decoys for each protein in making comparisons. The structures of WT azurin and the mutants were downloaded from the PDB (PDB ID: Apo, 1E65; WT, 4AZU; C112D/M121E at pH 7.0, 3NP3; C112D/M121E at pH 9.0, 3NP4; C121D, 3FQY; and C121D/M121L, 3FPY).^19,37−40^ In the crystal structures of WT and mutant azurins, copper is in the Cu(II) state. For the analysis of Cu(I) WT and mutants, we used the same crystal structures and replaced Cu(II) by Cu(I) in each case, followed by energy minimization and frustration analysis.

## Supporting Information Available

Coordinate parameters for Cu(I) and Cu(II) in the Rosetta function, comparison of crystal structure apo azurin frustration data with solution NMR data for the same protein, frustration patterns for apo- and copper-bound forms of WT azurin, structures of Cu(II)-binding sites in WT and mutant azurins, and changes in frustrated interactions in WT and mutant azurins when going from Cu(II) to Cu(I) (PDF)

## Notes

The input files and data from the frustration analysis in this work are deposited in GitHub: https://github.com/chemlover/azurin_frustration.

## Notes

The frustration analysis codes in this work are deposited in GitHub: https://github.com/chemlover/azurin_frustration.
