# Supplementary material for: Frustration Dynamics and Electron-Transfer Reorganization Energies in Wild-Type and Mutant Azurins
Source: J Am Chem Soc. 2022 Feb 16;144(9):4178–85. doi: 10.1021/jacs.1c13454 (PMC8915257; doi:10.1021/jacs.1c13454)
Supplement: Supplementary file 1 — ja1c13454_si_001.pdf [file ja1c13454_si_001.pdf]

Supporting Information

for

Frustration Dynamics and Electron Transfer Reorganization Energies in Wild Type and Mutant Azurins

Xun Chen[1,2], Mingchen Chen[3], Peter G. Wolynes[1,2,3,*], Pernilla Wittung-Stafshede[4,*], Harry B. Gray [5,*]

1. Center for Theoretical Biological Physics, Houston, TX, 77005, U.S.; 2. Department of Chemistry, Rice University, Houston, TX, 77005, U.S.; 3. Department of Biosciences, Rice University, Houston, TX, 77005, U.S.; 4. Department of Biology and Biological Engineering, Chalmers University of Technology, , 412 96 Gothenburg, Sweden; 5. Beckman Institute and Division of Chemistry and Chemical Engineering, California Institute of Technology, Pasadena, California 91125, U.S.;

**Correspondence:**	Peter G. Wolynes*	713-348-4101	pwolynes@rice.edu;
	Pernilla Wittung-Stafshede*	+46317728112	pernilla.wittung@chalmers.se;
	Harry B. Gray*	626-395-6500	hbgray@caltech.edu.

Content:

Tables S1-S2
Figures S1-S5
References

Table S1. The coordinate parameters of tetrahedral Cu(I) in Rosetta function, V1-V4 are virtual atoms to setup the geometry of tetrahedral Cu(I).

center atom	parameters			connected atom		
CU	0	0	0	CU	V1	V2
V1	0	0	2.2	CU	V1	V2
V2	0	70.5	2.2	CU	V1	V2
V3	120	70.5	2.2	CU	V1	V2
V4	120	70.5	2.2	CU	V1	V2

Table S2. The coordinate parameters of square planar Cu(II) in Rosetta function, V1-V4 are virtual atoms to setup the geometry of square planar Cu(II).

center atom	parameters			connected atom		
CU	0	0	0	CU	V1	V2
V1	0	0	1.000112	CU	V1	V2
V2	0	108	1.000112	CU	V1	V2
V3	0	0	1.000112	CU	V1	V2
V4	0	72	1.000112	CU	V1	V2

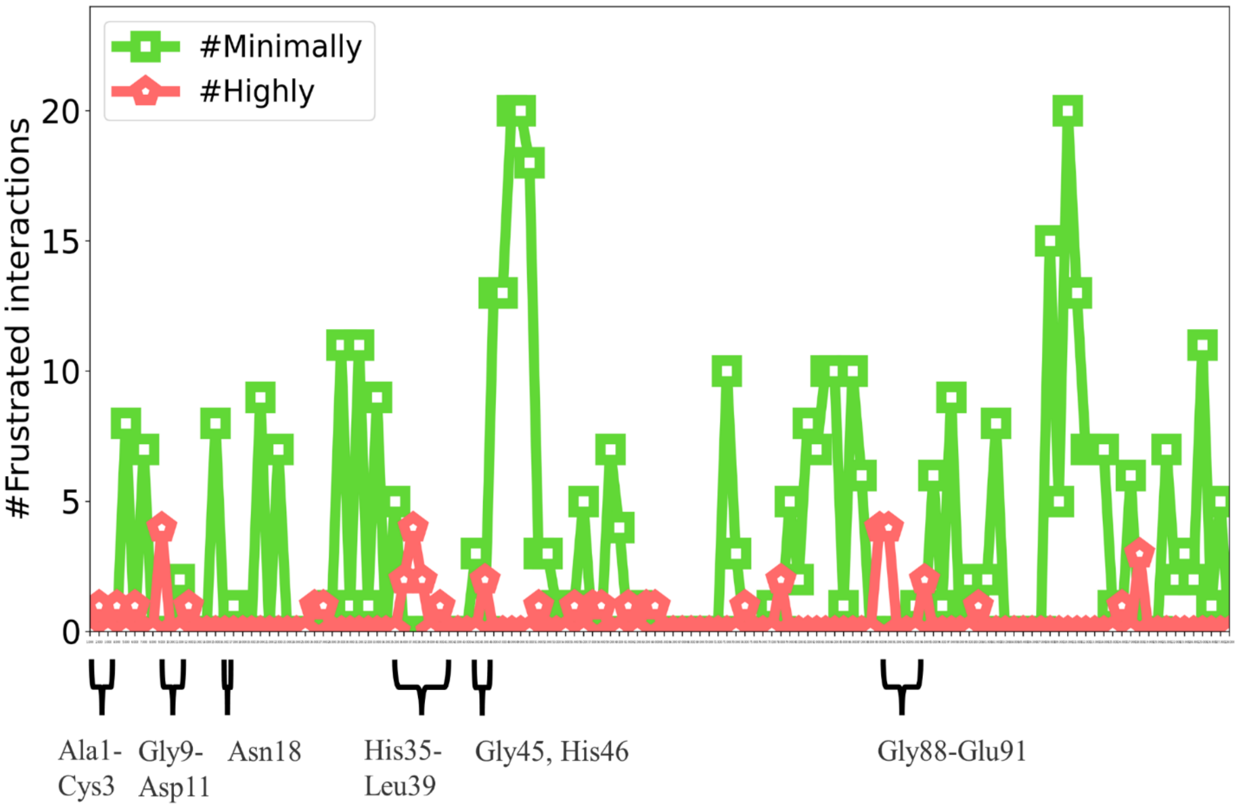

Figure S1. Number of (minimally and highly) frustrated interactions displayed per residue in WT apo azurin's sequence derived from frustration analysis on the crystal structure 1E65. Below the graph are indications for what residues (non-prolines) are missing in the $^{1}H,^{15}N$-HSQC spectrum of WT apo azurin in solution (1). The absence of signal in the NMR spectrum indicates involvement in intermediate exchange processes on the chemical shift timescale (i.e., residue possesses structural heterogeneity). There seems to be a correlation between apo protein data in solution and in the crystal structure: missing non-proline residues in the NMR spectrum possess more highly frustrated interactions and less minimally frustrated interactions than other residues in the frustration analysis of the apo protein crystal structure. Notably, of the missing residues in NMR, only two (Gly45, His46) belong to the copper binding site, the others are found in the copper binding site surroundings. Of those two, in our analysis, only Gly45 harbor highly frustrated interactions (but with residues outside the copper binding site).

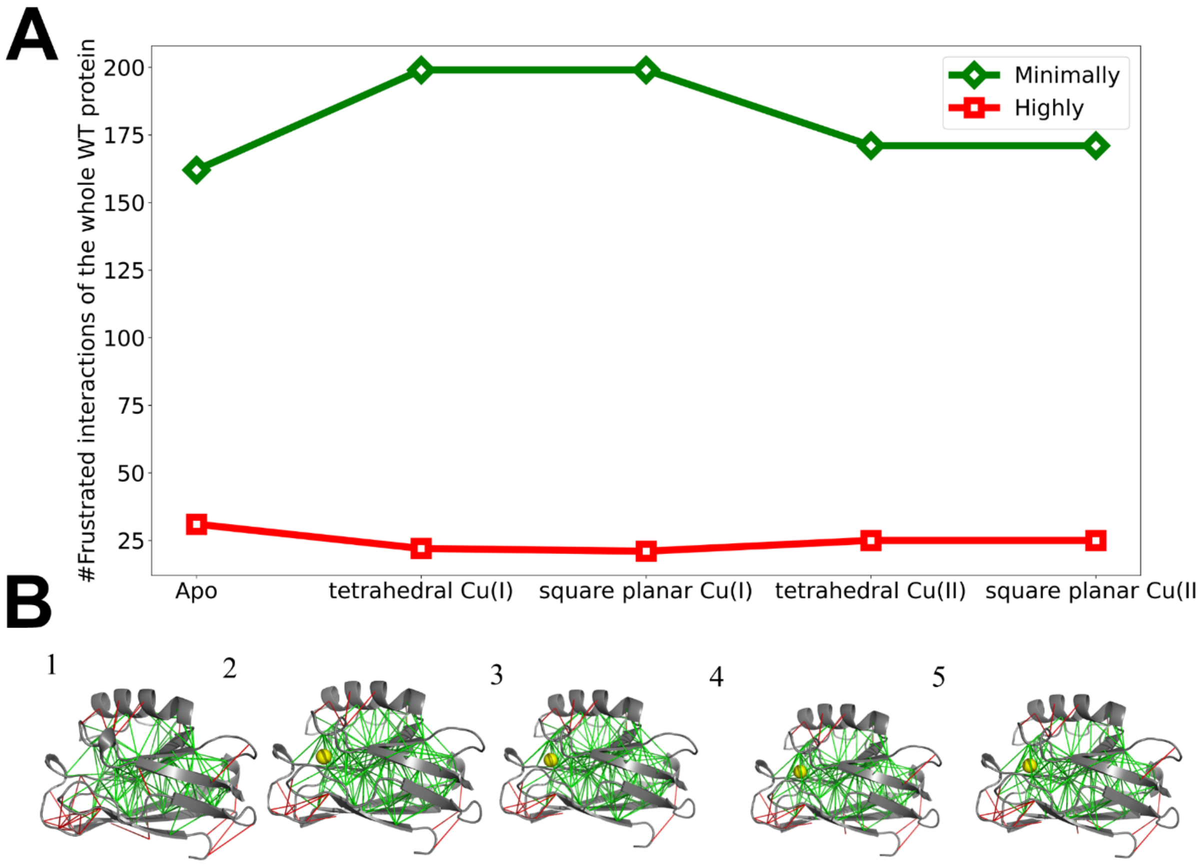

Figure S2. Frustration patterns of WT azurin in the apo form and different holo forms. A) The number of frustrated interactions in WT azurins on model tetrahedral and square planar copper centers. Color: Minimally, green; Highly, red; B) Atomistic frustration pattern in the apo form and different holo forms.1) Apo form. 2) tetrahedral Cu(I). 3) square planar Cu(I). 4) tetrahedral Cu(II), 5) square planar Cu(II), Colors: protein, gray; Cu, yellow sphere; the minimally frustrated interactions are shown as green lines; the highly frustrated interactions are shown as red lines.

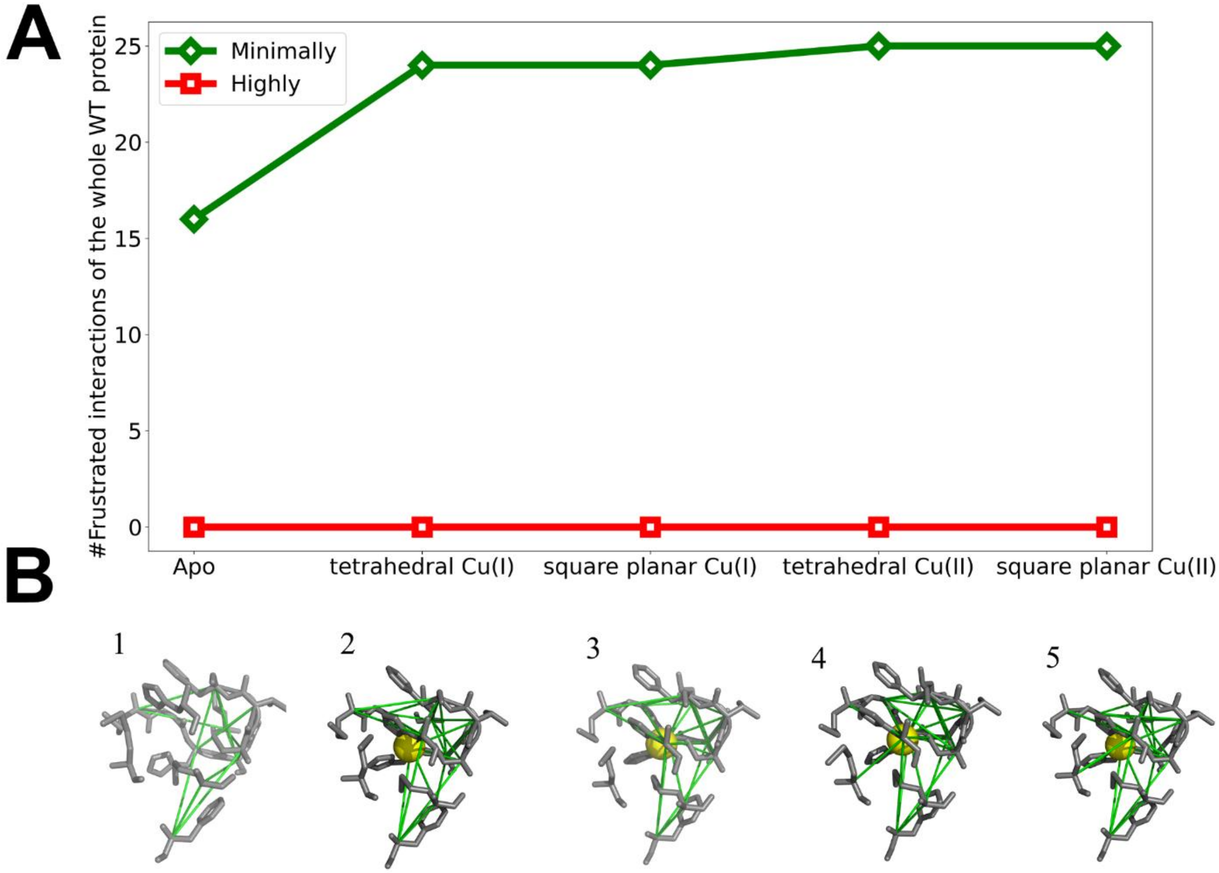

Figure S3. Binding pocket frustration pattern for WT azurin in the apo form and different holo forms. A) The number of frustrated interactions in binding pocket of WT azurins on model tetrahedral and square planar copper centers. Color: Minimally, green; Highly, red; B) Atomistic frustration pattern in the binding pocket of the apo form and different holo forms. 1) Apo form. 2) tetrahedral Cu(I). 3) square planar Cu(I). 4) tetrahedral Cu(II), 5) square planar Cu(II), Colors: pocket, gray; Cu, yellow sphere; the minimally frustrated interactions are shown as green lines; the highly frustrated interactions are shown as red lines.

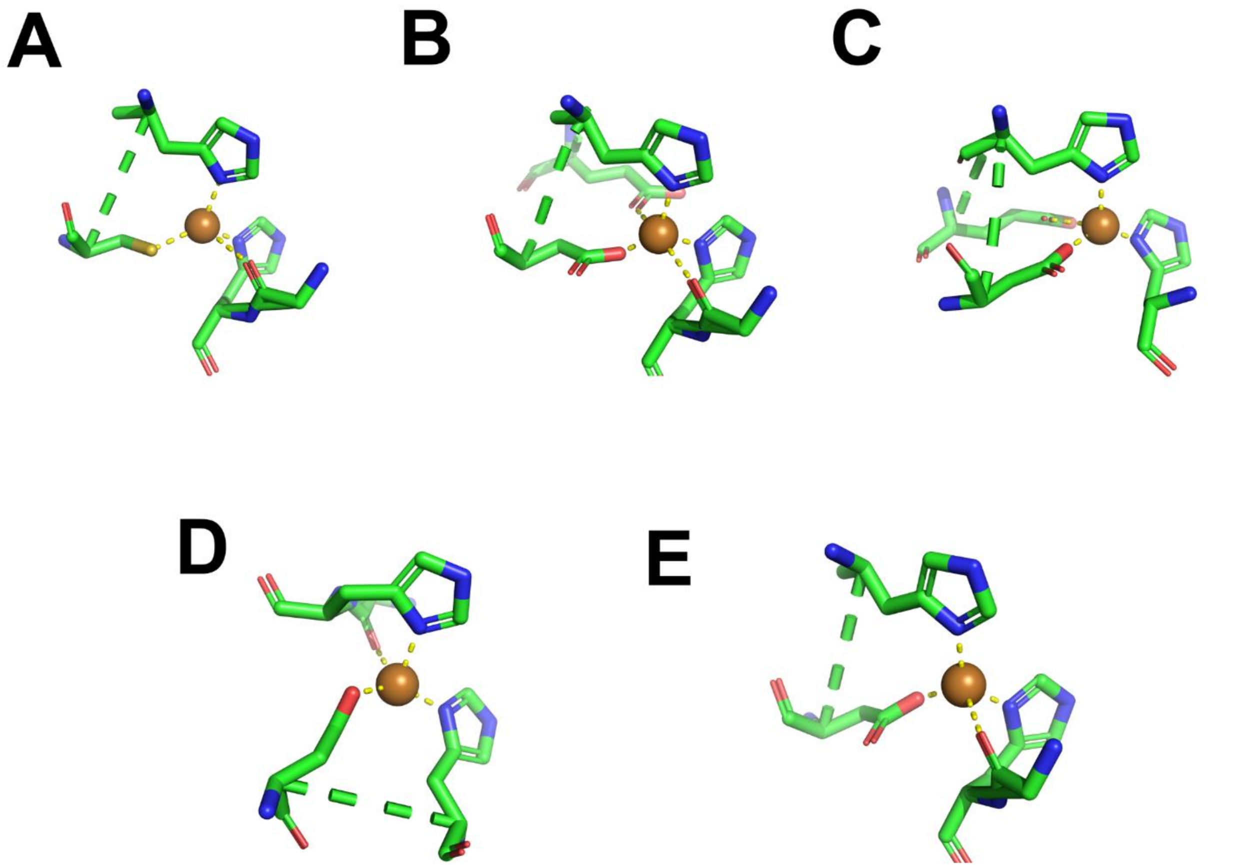

Figure S4. The Cu(II) center in the binding pocket of the experimental determined structures. A) WT azurin (PDBID: 4AZU); B) C112D/M121E at pH 7.0 (PDBID: 3NP3); C) C112D/M121E at pH 9.0 (PDBID: 3NP4); D) C112D/M121(PDBID: 3FQY); E) C112D/M121L (PDBID: 3FPY). Protein, sticks; Cu(II), brown sphere; nitrogen atoms, blue; oxygen atoms, red; carbon atoms, green; sulfur atoms, yellow. The interactions between Cu(II) and residues are shown as dashed lines in yellow.(2-5).

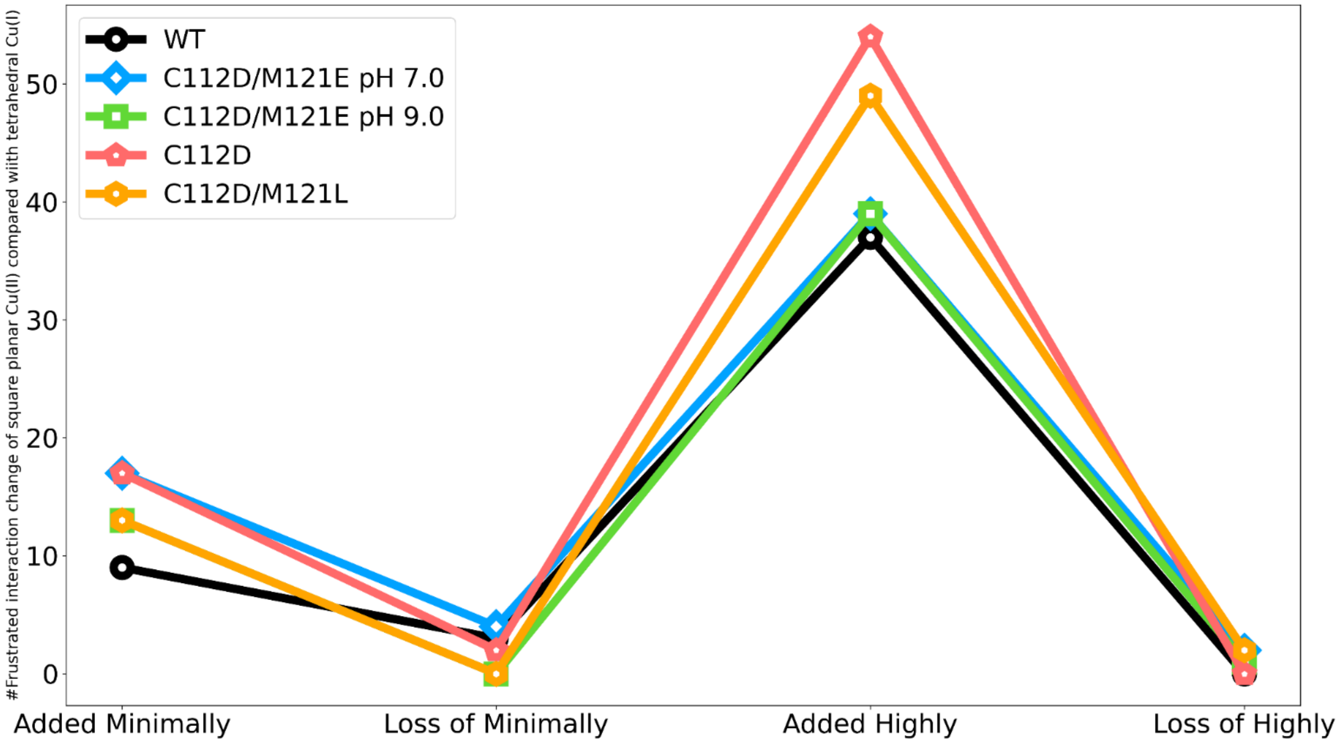

Figure S5. The change of frustrated interactions of square planar Cu(II) compared to tetrahedral Cu(I) for WT azurin and it mutants. Colors: WT, black; C112D/M121E at pH 7.0, blue; C112D/M121E at pH 9.0, green; C112D, red; C112D/M121L, orange. WT curve and C112D/M121L curve are overlapped

References

1. Zaballa, M.-E.; Abriata, L. A.; Donaire, A.; Vila, A. J. Flexibility of the metal-binding region in apo-cupredoxins. Proceedings of the National Academy of Sciences 2012, 109,9254–9259.9
2. Berman, H. M.; Westbrook, J.; Feng, Z.; Gilliland, G.; Bhat, T. N.; Weissig, H.; Shindyalov, I. N.; Bourne, P. E., The protein data bank. *Nucleic Acids Res.* **2000**, *28*, 235–242.
3. Nar, H.; Messerschmidt, A.; Huber, R.; van de Kamp, M.; Canters, G. W., Crystal structure analysis of oxidized Pseudomonas aeruginosa azurin at pH 5.5 and pH 9.0: A pH-induced conformational transition involves a peptide bond flip. *J. Mol. Biol.* **1991**, *221*, 765–772.
4. Lancaster, K. M.; George, S. D.; Yokoyama, K.; Richards, J. H.; Gray, H. B., Type-zero copper proteins. *Nat. Chem.* **2009**, *1*, 711–715.
5. Lancaster, K. M.; Sproules, S.; Palmer, J. H.; Richards, J. H.; Gray, H. B., Outer sphere effects on reduction potentials of copper sites in proteins: The curious case of high potential type 2 C112D/M121E Pseudomonas aeruginosa azurin. *J. Am. Chem. Soc.* **2010**, *132*, 14590–14595.
